# Spacer Loss upon 2D Ruddlesden–Popper Halide Perovskite Annealing Raises Film Properties and Solar Cell Performances

**DOI:** 10.3390/nano15100750

**Published:** 2025-05-16

**Authors:** Tao Zhu, Min Liu, Marie Cresp, Daming Zheng, Karol Vegso, Peter Siffalovic, Thierry Pauporté

**Affiliations:** 1 Institut de Recherche de Chimie Paris (IRCP), Chimie ParisTech, PSL Research University, CNRS, UMR8247, 11 rue P. et M. Curie, F-75005 Paris, Francemin.liu@chimieparistech.psl.eu (M.L.); marie.cresp@chimieparistech.psl.eu (M.C.); daming.zheng@chimieparistech.psl.eu (D.Z.); 2 Institute of Physics, Slovak Academy of Sciences, Dubravska Cesta 9, 84511 Bratislava, Slovakia; karol.vegso@gmail.com (K.V.); peter.siffalovic@savba.sk (P.S.); 3 Center for Advanced Materials and Applications (CEMEA), Slovak Academy of Sciences, Dubravska Cesta 5807/9, 84511 Bratislava, Slovakia

**Keywords:** Ruddlesden–Popper halide perovskite, precursor solution composition, phenylmethylammonium, deprotonation and volatilization, solar cells

## Abstract

Using reduced-dimensional halide perovskites is emerging as a promising strategy for enhancing the stability of optoelectronic devices such as solar cells, even if their performances remain a step below those of the 3D halide perovskites. Two-dimensional Ruddlesden–Popper (2D-RP) structures are characterized by the *n* parameter that represents the number of PbI_6_ layers in the spacer-separated perovskite slabs. The present study focuses on formamidinium (FA)-based 2D-RP type perovskites denoted as PMA_2_FA_*n*−1_Pb_*n*_I_3*n*+1_ (PMA = Phenylmethylammonium or benzylammonium). We investigate the effect of *n* on the one step growth mechanism and the film morphology, microstructure, phase purity, and optoelectronic properties. Our findings demonstrate that the average *n* is not only determined by the initial spacer content in the precursor solution but also by the thermal annealing process that leads to a partial spacer loss. Depending on *n*, perovskite solar cells achieving a power conversion efficiency up to 21%, coupled with enhanced film stability compared to 3D perovskites have been prepared. By using MACl additive and an excess of PbI_2_ in the perovskite precursor solution, we have been able to achieve high efficiency and to stabilize the *n* = 5 perovskite solar cells. This research represents a significant stride in comprehending the formation of FA-based layered perovskites through one-step sequential deposition, enabling control over their phase distribution, composition, and orientation.

## 1. Introduction

Metal halide perovskites featuring organic–inorganic components have garnered significant attention due to their remarkable opto-electronic properties, such as a tunable bandgap, good properties for charge carrier generation and transport, high charge carriers mobility, a facile preparation from solutions and cost-effective manufacturing [1,2]. Typically, 3D organic–inorganic halide perovskites adhere to the ABX_3_ chemical formula, where A represents an organic cation (e.g., methylammonium (MA^+^) or formamidinium (FA^+^)) or Cs^+^, B is a divalent metal cation (e.g., Pb^2+^, Sn^2+^, or Ge^2+^), and X is a halide anion (typically Cl^−^, Br^−^, I^−^, or their combinations) [3,4]. In the three-dimensional (3D) perovskite framework, [BX_6_]^4−^ octahedra are connected through their corner [5], and are counterbalanced by an equivalent number of positively charged cations occupying the confined cavities formed by the metal halide network [6]. Introducing large organic cations (called spacer) leads to a decrease in dimensionality and produces lamellar structures where perovskite slabs are separated by layers of spacer cations [7,8,9]. Reducing the dimensionality of the halide perovskite is supposed to confer a higher stability due to the introduction of stabilizing large cations. It results in a higher crystal formation energy [2,9].

2D Ruddlesden–Popper (2D-RP) halide perovskites, with the general formula A’_2_A_*n*−1_B_*n*_X_3*n*+1_, were first employed in solar cells by introducing phenylethylammonium (PEA^+^) into MAPbI_3_ perovskite. This resulted in the transformation of the 3D structure into a 2D one (PEA)_2_(MA)_2_Pb_3_I_10_ (*n* = 3) [10]. Although perovskite solar cells (PSCs) utilizing this targeted material exhibited a power conversion efficiency (PCE) of only 4.7%, the films exhibited an enhanced stability under room temperature and an ambient relative humidity (RH) of 52% exposure [10]. Following this success, significant advancements have been made through solution-processing techniques, molecular chemistry, and a large understanding of the physical properties of the films [11,12,13,14]. The crystalline quality of the final film depends on the formation of intermediate phases, notably involving solvents, which have a composition that must be controlled to lead to a pure final phase after thermal annealing [11,12,13,14].

Unlike the well-explored MA-based layered 2D perovskites, FA-based 2D perovskites have been less studied. Effective strategies for tailoring energy characteristics to stabilize the 3D α-FAPbI_3_ phase in challenging environmental and operational conditions, remain to be determined [15]. FAPbI_3_-based systems present an inherent phase instability and require mixing with smaller cations for stabilization. The challenges associated with producing high-quality and stable FA-based layered perovskite films compared to their MA-based analogs are also linked to the fate of additives incorporated in the perovskite precursor solution [16]. FAPbI_3_ δ-phase featuring a hexagonal (face-sharing) one-dimensional (1D) structure is favored at room temperature due to its higher thermodynamic stability compared to the photoactive FAPbI_3_ α-phase, characterized by a cubic 3D corner-sharing [MX_6_]^4−^ octahedral framework [17]. Consequently, when fabricating FA-based 2D layered perovskites, both nucleation and growth mechanisms, whether involving corner-sharing or face-sharing structures, must be considered [18]. This complexity of crystallization for FA-based 2D layered perovskites contrasts with the more straightforward process observed in MA-based analogs [19]. FA^+^ exhibits lower interaction with the dimethyl sulfoxide (DMSO) solvent in comparison to MA^+^, resulting in a diminished tendency to form a precursor–solvent intermediate phase during film formation [20]. In the search for stable and efficient PSC based on 2D-RP, the preparation of homogeneous phase pure layers is an important point [21,22,23]. Another one is the employment of MACl as an additive in the production of 2D-RP perovskite layers efficient in diverse optoelectronic devices, including solar cells and light-emitting diodes [24,25,26]. MACl plays a pivotal role in the production of perovskite materials by enhancing film quality, improving stability, enabling property tuning, and reducing defects [27,28].

The objective of this paper is to provide a comprehensive understanding of the effect of the *n* parameter on the growth process of 2D-RP type PMA_2_FA_*n*−1_Pb_*n*_I_3*n*+1_ (PMA^+^ being phenylmethylammonium, also called benzylammonium) perovskite films prepared via one-step deposition. Compositional, structural, and optoelectronic changes in the layers have been investigated. The layers have also been applied in PSC. We synthesized a series of 2D-RP perovskite films starting from perovskite precursor solutions (PPSs) with stoichiometries corresponding to a general molecular formula of PMA_2_FA_*n*−1_Pb_*n*_I_3*n*+1_ with *n* = 1, 2, 3, 5, 9, and 100. We have investigated the effect of MACl additives and quantified the effect of *n* on the incorporation of MA^+^ in the structure. ^1^H NMR has also been used to show that the PMA^+^ spacer content in the layer not only depends on the initial *n* in the PPS but also on a partial loss of PMA^+^ upon the thermal annealing. The *n* value in the final layer is markedly increased. We have determined that suitable *n* values can yield solar cells with good stability and efficiency.

## 2. Results and Discussion

### 2.1. Characterizations of the Perovskite Films

The FA-based 2D-RP perovskite layers were synthesized using the one-step preparation method presented in Figure S1a. As a pivotal additive for the preparation of high-quality halide perovskite layers, we first investigated the use of MACl additive for the preparation of 2D-RP layers. We selected *n* = 9 to investigate the optimal MACl content in PMA_2_FA_*n*−1_Pb_*n*_I_3*n*+1_ perovskite precursor solutions (PPSs). The morphologies of these samples (30% MACl, 36% MACl, and 45% MACl of FAI content in the PPS) were examined by scanning electron microscopy (SEM). The images are reported in Figure S1b–d. With 30% MACl additive, the perovskite grain size was relatively small (approximately 500 nm) and the surface was rather rough, which is unfavorable for the charge carrier transport in perovskite. With 36% MACl additive, the perovskite grain size was enlarged to about 1000 nm, and the surface became smoother and denser. Such a morphology is known to promote effective charge carrier transport in perovskite. When 45% MACl additive was employed, the perovskite grain size remained unchanged, but noticeable pinholes appeared, potentially leading to voltage loss and leakage current. These pinholes may result from inappropriate film growth. We therefore retained 36% MACl of additive in the rest of the study as the most conducive for the growth of perovskite 2D RP layers.

**Figure 1:**
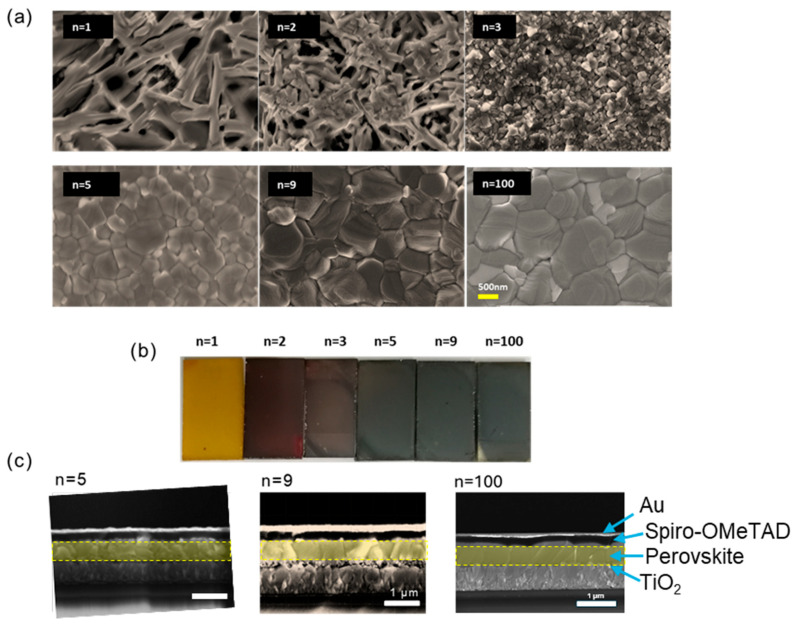
(**a**) SEM top views of the morphologies of perovskite films with PMA_2_FA_*n*−1_Pb_*n*_I_3*n*+1_ (*n* = 1, 2, 3, 5, 9, and 100) initial composition. (**b**) Pictures of the perovskite layers. (**c**) Cross-sectional SEM images of *n* = 5, 9, and 100 films.

The effect of *n* was then studied for various PMA_2_FA_*n*−1_Pb_*n*_I_3*n*+1_ precursor compositions. The SEM analysis in Figure 1a explores the morphologies of these specimens prepared for *n* = 1, 2, 3, 5, 9, and 100. The morphology of the 2D perovskite layers, encompassing characteristics such as crystallinity, grain size, smoothness, and pore presence, significantly impacting the performance of perovskite solar cells. The *n* = 1 sample exhibits a staggered branch-like structure. For *n* = 2, the branch-like structure diminishes, and some small cubic grains emerge. With *n* = 3, only small grains with an average size of 150 nm are evident from a top view. In the case of *n* = 5, the grain size increases to an average of 800 nm, and the surface becomes notably compact and smooth. *n* = 9 and *n* = 100 result in large grain sizes approaching 1 µm. No PbI_2_ crystals were observed on the surface. The substantial grain size observed at high *n* can be attributed to the chloride additive (MACl), which facilitates the perovskite grains formation and growth. The overall layer appearance indicates a color transition in the samples from orange to red and then to black with an increasing *n* value (Figure 1b). The cross-section images show that the *n* = 9 and *n* = 100 samples exhibit large grains, with their thickness matching the perovskite layer, as illustrated in Figure 1c. The targeted morphology obtains each crystal grain in contact with the hole-transporting layer on one side and the electron-transporting layer on the other side in solar cells.

**Figure 2:**
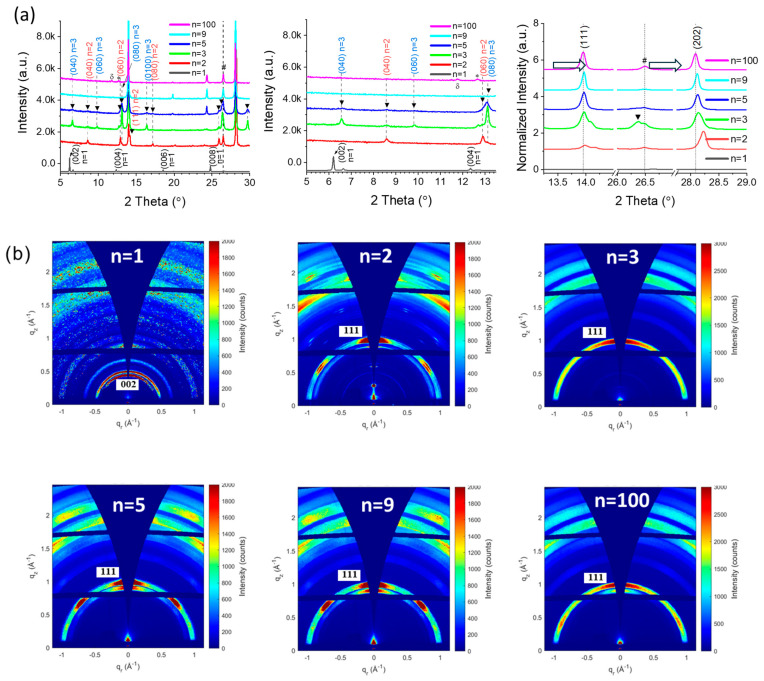
(**a**) θ-2θ XRD patterns of PMA_2_FA_*n*−1_Pb_*n*_I_3*n*+1_ (*n* = 1, 2, 3, 5, 9, and 100) 2D perovskite layers. The two figures on the right display zoomed XRD patterns for clarity. * corresponds to PbI_2_ and # corresponds to FTO. (**b**) GIWAXS patterns of the PMA_2_FA_*n*−1_Pb_*n*_I_3*n*+1_ (*n* = 1, 2, 3, 5, 9, and 100) 2D perovskite layers measured at 1° incidence angle.

θ-2θ X-ray diffraction (XRD) was employed to characterize the perovskite films, as illustrated in Figure 2a. The peaks indexation shows that the *n* = 1, *n* = 9, and *n* = 100 exhibited good phase purity. The *n* = 2, 3 and 5 exhibited mixed peaks indexed to *n* = 2, *n* = 3, and high-*n* phases. These 2D perovskite films were composed of a mixture of phases and were not phase pure. All the 2D perovskites with FA^+^, starting from *n* = 2, included the high-*n* phases. We also observed that, as *n* values decreased, the (111) and (202) peaks shifted to the right (Figure 2a). It indicates a lattice shrinking of the 2D high-*n* phase perovskite, which is assigned below to enrichment in the MA^+^ cation. In (PMA)_2_PbI_4_ perovskite films with *n* = 1, two peaks at 6.18° and 12.36° are observed, corresponding to the (002) and (004) crystal planes of *n* = 1, respectively. In (PMA)_2_FAPb_2_I_7_ perovskite films with *n* = 2, the diffraction peaks at 8.6°, 12.9°, and 14.2° are indexed as the (040), (060), and (111) crystal planes of *n* = 2 phase, respectively. For (PMA)_2_FA_2_Pb_3_I_10_ (*n* = 3), three diffraction peaks at 6.57°, 9.83°, and 13.14° correspond to the (040), (060), and (080) crystal planes of *n* = 3 phase, respectively. Another peak near 13.96° corresponds to the (111) crystal plane of the high-*n* phase. In (PMA)_2_FA_4_Pb_5_I_16_ (*n* = 5), there are three peaks at 6.57°, 9.83°, and 13.14° corresponding to the (040), (060), and (080) crystal planes of *n* = 3 phase, and one peak at 8.6° assigned to the (040) crystal plane of *n* = 2 phase. Except for *n* = 1, all PMA_2_FA_*n*−1_Pb_*n*_I_3*n*+1_ exhibit high-*n* phases (near 14°). The XRD patterns of the PMA_2_FA_*n*−1_Pb_*n*_I_3*n*+1_ samples with *n* = 9 and *n* = 100 exhibit peaks corresponding to (111) plane.

Grazing-incidence wide-angle X-ray scattering (GIWAXS) measurements were carried out to further investigate the effect of the *n* value on the crystallinity and orientation of quasi-2D (q2D) perovskite (Figure 2b). The *n* = 1 sample exhibits diffraction rings, indicating the absence of preferential orientation [29,30]. In contrast, the *n* = 2, *n* = 5, *n* = 9, and *n* = 100 samples revealed a textured layer, as a (111) maximum along the q_z_ direction indicates a vertical orientation of 2D perovskite slabs PMA_2_FA_*n*−1_Pb_*n*_I_3*n*+1_ with the (111) planes parallel to the sample surface [30]. It is important to note that the (202) diffraction visible in Figure 2a is masked by an inaccessible area of GIWAXS geometry. With the exception of the sample with *n* = 3, all the others also exhibit an additional (111) maximum at approximately 45° from the normal direction (q_z_), indicating the presence of a rotated phase, also termed corner-up orientation [7].

**Figure 3:**
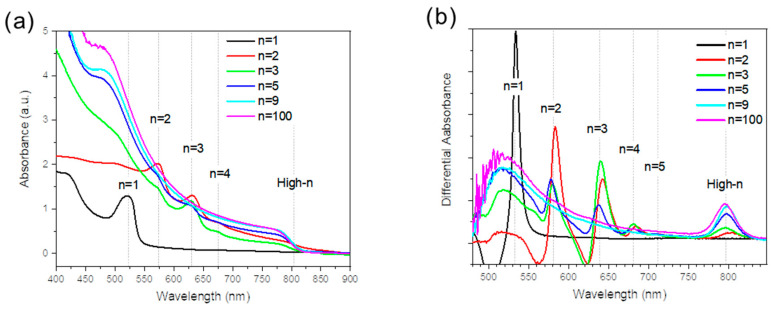
(**a**) Spectra of UV-Vis absorbance for the 2D perovskite PMA_2_FA_*n*−1_Pb_*n*_I_3*n*+1_ (*n* = 1, 2, 3, 5, 9, and 100) layers. (**b**) Differential absorbance of (**a**) spectra.

The UV-Vis absorbance spectra of the films were measured (Figure 3a) and the spectra were derived to precisely localize the peak positions (Figure 3b). They were found at 532 nm, 582 nm, 640 nm, 682 nm, 716 nm, and 802 nm and are attributed to PMA_2_FA_*n*−1_Pb_*n*_I_3*n*+1_ phases, with *n* = 1, *n* = 2, *n* = 3, *n* = 4, *n* = 5, and high-*n* phases, respectively. The absorbance of the high-*n* peaks in this series increased with the *n* values. Samples of *n* = 1, *n* = 9, and *n* = 100 exhibited a single absorption peak at approximately 535 nm, 805 nm, and 806 nm, respectively. It indicates pure phases and is consistent with the XRD data. Conversely, 2D perovskite films with 2 ≤ *n* ≤ 5 exhibited mixed phases. For example, the sample prepared with the initial *n* = 2 solution stoichiometry included *n* = 2, *n* = 3, *n* = 4, and high-*n* phases in the differential illustration of the absorbance curves (Figure 3b).

**Figure 4:**
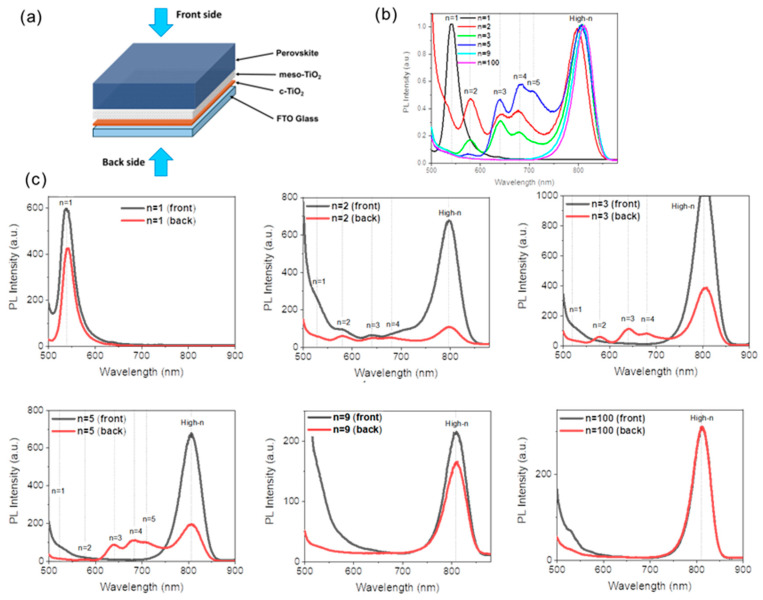
(**a**) Schematic representation of the back side and front side excitations for PL measurements of perovskite layers. (**b**) Steady-state PL spectra of 2D perovskite films PMA_2_FA_*n*−1_Pb_*n*_I_3*n*+1_ (*n* = 1, 2, 3, 5, 9, and 100) acquired from the back side. (**c**) PL spectra of 2D perovskite films PMA_2_FA_*n*−1_Pb_*n*_I_3*n*+1_ (*n* = 1, 2, 3, 5, 9, and 100) measured from the back and front sides.

The films were further investigated by measuring their steady-state photoluminescence (PL) spectra shining the sample from the back side and the front side (Figure 4a). In Figure 4b, mixed 2D PL peaks of *n* = 2, *n* = 3, *n* = 4, *n* = 5 phases are found for *n* = 2, *n* = 3, and *n* = 5. Samples are measured from the back side. The results are in agreement with the XRD and UV-Vis measurements. Moreover, the high-*n* PL peaks were blue-shifted from 812 nm to 797 nm as the *n* values decreased as expected for quasi-2D perovskite [31]. To assess the spatial distribution of these 2D phases, we collected the steady-state PL spectra under two distinct excitation configurations: from the front side (perovskite side) and the back side (glass side) (Figure 4a); the results are presented in Figure 4c. For an initial *n* = 5 stoichiometry, the high-*n* emission peak was observed at 805 nm, regardless of the excitation side. Generally, the low-*n* 2D emission peaks were absent or of smaller intensity for the front side excitation. The inhomogeneous distribution is a common occurrence in 2D phases of perovskite films. However, the fact that hydrophobic quasi-2D perovskites are mainly distributed at the bottom of the perovskite layer restricts the expected stability improvement. Layers prepared from *n* = 1 PPS are phase pure, and those prepared from *n* = 9, and 100 PPSs present only the high-*n* phase and are homogeneous.

### 2.2. Composition of the Layer

The constituents in the precursor solution included PMAI, FAI, MACl, and PbI_2_ dissolved in a DMF/DMSO solvent mixture. While certain components are eliminated upon the layer preparation, especially upon thermal annealing (TA), it is important to follow the composition of the perovskite layers upon their preparation. The Cl content was quantified using the GD-OES technique. The resulting evolution of Cl profile upon annealing is presented in Figure S2a. The as-spin coated layer is rich in Cl. We observed a homogeneous elimination of Cl upon TA. Figure S2b shows that the Cl amount homogeneously decreases upon the TA, with the majority of Cl being eliminated within the first 2 min. Only traces remained after the annealing completion.

**Figure 5:**
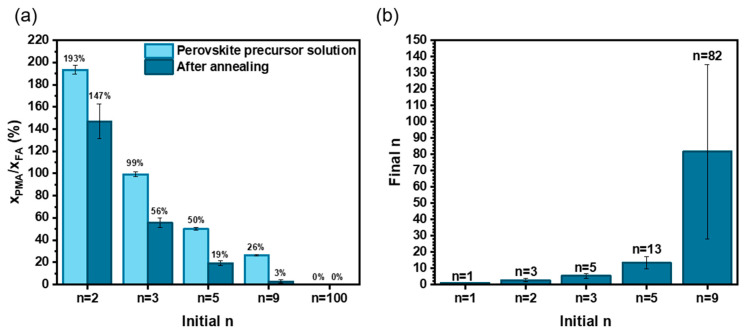
^1^H NMR titration of organic cations. Films prepared with 36 mol% MACl additive. (**a**) Ratio of PMA/FA in the PPS (light) and in the perovskite films after full annealing at 150 °C for 15 min (dark). (**b**) Final *n* value in the perovskite PMA_2_FA_*n*−1_Pb_*n*_I_3*n*+1_ versus the initial *n* in the PPS.

The organic cations present in the fully annealed perovskite films were titrated by ^1^H NMR after the dissolution of layers in deuterated DMSO-d_6_ solvent. The A-site and spacer organic cations were identified and titrated from the spectra. The results of quantification are given in Figure S3. The quantity of PMA^+^, MA^+^, and FA^+^ cations in annealed perovskite films was determined by ^1^H NMR spectra analysis. We have also detected traces of MFA^+^ (3-methyl formamidinium) produced by the reaction between MA° (the deprotonated MA^+^) and FA^+^ [32]. The results of quantification of the various samples are disclosed in Figure S3. All the samples contained MA^+^, and we can conclude that the A-sites were filled by both MA^+^ and FA^+^. Figure S4 compares the molar ratio between (MFA^+^ + MA^+^) and FA^+^. Above *n* = 3, we observe a decrease in MA^+^ content with *n*, with more MA^+^ fraction elimination. Figure 5a compares the PMA^+^/FA^+^ molar ratio between the PPS and the final fully annealed layer. It can be seen that PMA^+^ content is lower in the final film compared to the PPS. As most of FA^+^ is supposed to remain in the layer, we can deduce that there is a partial spacer elimination upon annealing. PMA^+^ is partially deprotonated and forms volatile PMA° [33]. Finally, we determined the average *n* value in the final layer [33]. Figure 5b compares the final average *n* and the initial *n* in the PPS. It is noteworthy that, when A-site cations are present in the PPS, the final average *n* is higher than the initial one. The final average *n* in the fully annealed perovskite films with *n* = 1, 2, 3, 5 and 9 in the PPS are *n* = 1, 3, 5, 13 and 84, respectively. The discrepancy increases with the initial *n* value. For clarity, in the rest of the paper, we retain the initial *n* for the sample notation, keeping in mind that the true final *n* is higher.

### 2.3. Layer Formation Process

**Figure 6:**
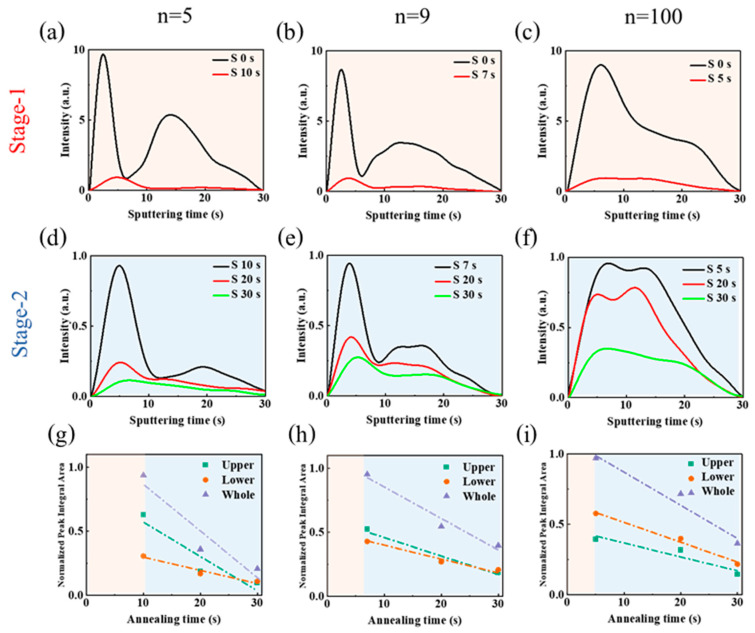
Sulfur element (S) GD-OES profile measured during the thermal annealing. The progress of perovskite precursor films is monitored during Stage-1 and Stage-2 of thermal annealing. (**a**) *n* = 5, (**b**) *n* = 9, and (**c**) *n* = 100 PMA_2_FA_*n*−1_Pb_*n*_I_3*n*+1_ films at Stage-1. (**d**) *n* = 5, (**e**) *n* = 9, and (**f**) *n* = 100 PMA_2_FA_*n*−1_Pb_*n*_I_3*n*+1_ films at Stage-2. Normalized peak integral area of the GD-OES S profiles for the upper layer, lower layer, and the whole film at different annealing times: (**g**) *n* = 5, (**h**) *n* = 9, and (**i**) *n* = 100 PMA_2_FA_*n*−1_Pb_*n*_I_3*n*+1_ films. (Green dashed line: linear fit line of the upper layer at Stage-2; red dashed line: linear fit line of the lower layer at Stage-2; blue dashed line: whole film at Stage-2).

We have shown in the previous section that strong changes occur in the films upon the thermal annealing process. We focus here on the film formation process for *n* = 5, *n* = 9 and *n* = 100 initial compositions. The changes occurring upon layer thermal annealing were followed by using the glow discharge optical emission spectroscopy (GD-OES) technique (Figure S5a, Supporting Information). GD-OES provides the elemental profiles of the layers since the sputtering time (x-axis) is related to the depth of the probed sample. By measuring the samples at increasing thermal annealing time, changes in the element profiles can be followed. We started from the humid precursor films obtained by spin-coating [34,35]. DMSO remains present in the film as residual solvent bounded to Pb^2+^. Its elimination behavior gives crucial information about the layer recrystallization and on the quasi-2D perovskite layer formation. We have analyzed the quasi-2D perovskite layers results on the basis of our prior exploration of 3D perovskite film formation [36,37]. DMSO contains sulfur element (S) that can be tracked by the GD-OES technique. Figure 6 shows S element concentration profiles (until reaching the TiO_2_ basal layers) for various initial *n* quasi-2D perovskite layers and for increasing annealing times. Figure S5 demonstrates that the meso-TiO_2_ layer is attained after about 30 s of sputtering time. In the as-spin coated layer, with no thermal annealing, the DMSO profile exhibits a peak in the upper part of the *n* = 5 and *n* = 9 layers (Figure 6a,b). Strikingly, the solvent distribution is more uniform for the initial *n* = 100 layer (Figure 6c).

**Figure 7:**
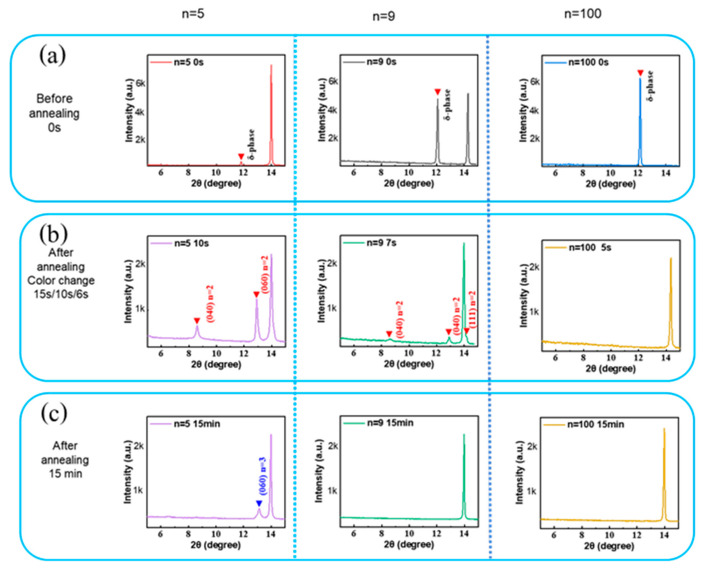
The θ-2θ XRD pattern of the *n* = 5, 9, and 100 films prepared by spin-coating, and annealed for various times: (**a**) as-spin coated, (**b**) at color change, and (**c**) after full annealing.

Based on the layers’ visual aspect change upon the annealing process, two distinct stages have been defined, as shown in Figure S6. XRD patterns of the initial, as-coated, films show that low-*n* favors the formation of α-phase perovskite. It decreases with *n* while the δ-phase increases with *n* (Figure 7a). In Stage-1, the solvent is rapidly eliminated, as evidenced by GD-OES profiles, where the sulfur peak localized in the upper layer part disappears for the *n* = 100 sample and significantly decreases for *n* = 5 and *n* = 9. During Stage-1, a quasi-2D perovskite phase (mainly *n* = 2) forms through the precursor phase decomposition (Figure 7b) [38].

The perovskite phase grains experience selective growth in a specific direction during Stage-2, while the residual solvent continues to be eliminated. The evolution of the solvent profile upon its gradual elimination in Figure 6d–f provides information on the perovskite grain growth speed and direction. In Figure 6d, GD-OES results for *n* = 5 perovskite layers show that DMSO is preferentially eliminated near the surface. The faster elimination of the solvent from the upper part of the layer is the signature of a downward growth of grains from the top to the bottom. This favors the development of multiple and oblique grain boundaries, as well as relatively small quasi-2D perovskite grains (Figure 1c). The *n* = 9 sample (Figure 6e) exhibits an S profile change similar to the *n* = 5 sample. The elimination is still faster at the top but the speed gap between the top and the bottom is reduced. This results in the formation of a perovskite layer with bigger grains (Figure 1c). In the *n* = 100 sample, the S profiles exhibit distinctive characteristics (Figure 6f). The S profile is distributed more uniformly throughout the film thickness and during annealing, the solvent is eliminated uniformly from the layer thickness. This indicates that lateral grain growth occurs, leading to the formation of uniform perovskite layers with large monolithic grains, as demonstrated in the cross-sectional SEM image (Figure 1c).

For a further analysis, we partitioned the film into an upper and a lower part, utilizing the methodology and quantitative tools described in our Ref. [36], and illustrated in Figure S7. Figure 6g–i provides the integration of the GD-OES curves upon thermal annealing for the upper and the lower layers upon Stage 2. From the curve’s slopes, related to the solvent elimination speed, we have calculated the GI(%) parameter. The slope values and calculated GI(%) values are gathered in Table 1.

**Figure 8:**
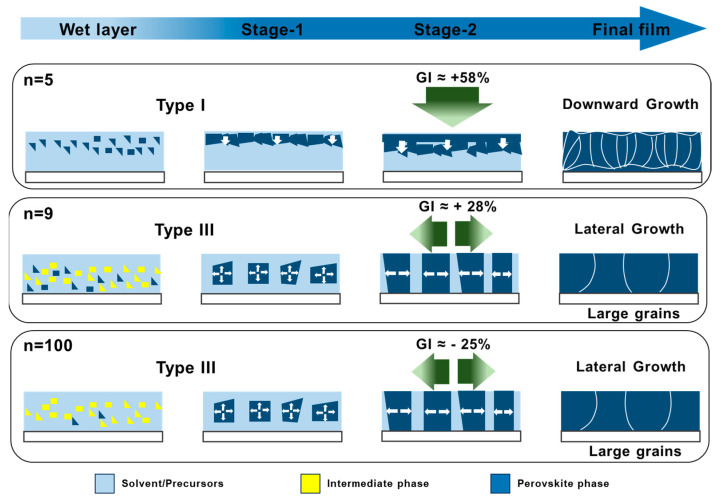
Schematic process of the growth of perovskite layers upon thermal annealing for various initial *n* values. The figure illustrates the effect of the initial *n* on the nucleation, recrystallization, crystal growth, and final layer morphology, as well as the GI parameter and the growth type. The green arrows denote the orientations of the growth.

From Figure 6 and Table 1 and based on our previous work [36], the GI value higher than 30% for *n* = 5 corresponds to a downward growth (Type I). For this growth, solvent elimination is difficult, resulting in the formation of multiple and oblique grain boundaries and yielding relatively small perovskite grains. Furthermore, these layers manifest higher roughness compared to films generated through lateral growth. Conversely, the slopes for the upper and lower layers of *n* = 9 and *n* = 100 samples are close (−30% < GI(%) < +30) and the growth is of type III. It results in the formation of uniform layers with large grains and a monolithic structure as the growth is lateral [36]. It generates a monolithic structure with diminished and vertically oriented grain boundaries, while the films are smoother (Figure 1c). The monolithic structure must be targeted to achieve fast charge transport and collection at the selective contacts [35]. This study shows that varying values of *n* regulate solvent elimination, thereby exerting control over the development of the perovskite layer across its entire depth. The values *n* = 9 and *n* = 100 enhance the morphological and structural quality of the layers. These results are summarized in Figure 8.

### 2.4. Characterization of the Solar Cells

**Figure 9:**
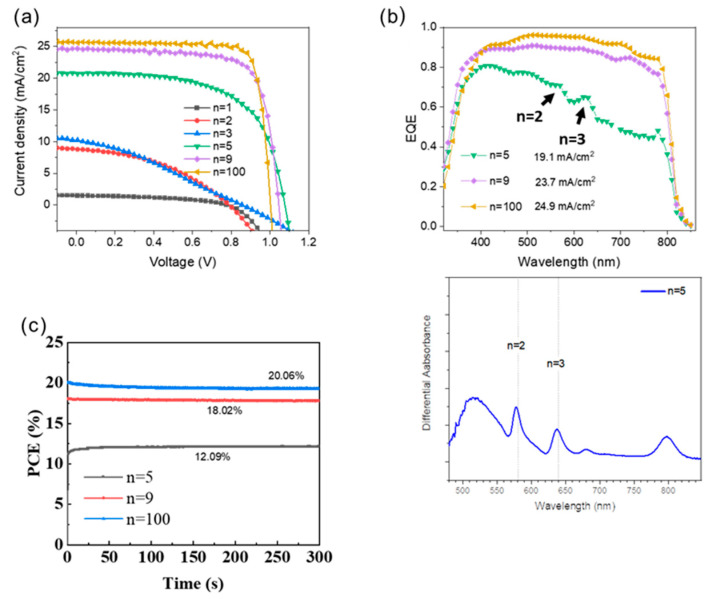
Effect of *n* parameter on the (**a**) *J-V* reverse scan curves, (**b**) EQE plots, and differential UV-Vis absorbance. (**c**) Normalized PCE at the maximum power point tracking curve of PSCs under standard AM 1.5G illumination in air at 25 °C with 50–55% RH.

The perovskite films were used to prepare glass/FTO/c-TiO_2_/meso-TiO_2_/perovskite/Spiro/Au solar cells with c-TiO_2_ being a compact TiO_2_ layer prepared by spray pyrolysis and meso-TiO_2_ a mesoporous layer of TiO_2_. Typical *J-V* curves and the determined photovoltaic parameters as a function of *n* are reported in Figure 9a, and in Table 2, respectively. The photocurrents and efficiencies increased with the *n* values as the high-*n* phase gradually gained dominance in the structure. The increase in open circuit voltage (V_OC_) from *n* = 1 to *n* = 5 can be attributed to the reduction in interface recombination and shunting with the decline of the branch-like structure and the layer densification. From *n* = 5 to *n* = 100, the V_OC_ slightly decreased. It is attributed to the reduction in the perovskite bandgap. The most efficient 2D perovskite solar cell was measured for *n* = 100, with a *V_OC_* of 1.023 V, a *J_SC_* of 25.68 mA/cm^2^, an *FF* of 80.56, and a *PCE* of 21.16%. For *n* = 9 the PSC exhibited a *V_OC_* of 1.022 V, a *J_SC_* of 24.72 mA/cm^2^, *FF* of 76.72, and *PCE* of 19.38%. Remarkably, the *PCE* of this study is high for a PMA-based *n* <10 2D halide perovskite devices, demonstrating its competitiveness [38,39]. The low-*n* PSCs showcased their maximum *PCE* on the reverse scan at 0.53%, 2.88%, 2.96%, and 13.78% for *n* = 1, 2, 3, and 5, respectively.

The efficiency of photocurrent generation versus the wavelength was assessed through EQE spectra measurements (Figure 9b). The integrated *J_SC_* values are 19.1, 23.7, and 24.9 mA/cm^2^ for *n* = 5, *n* = 9, and *n* = 100, respectively. They are in good agreement with the measured J_SC_. For *n* = 5, the EQE spectrum shows peaks which match with the UV/Vis absorption spectrum (Figure 3a) and are assigned to the *n* = 2 and *n* = 3 2D phases (Figure 9b). To further illustrate the photoelectric performance of *n* = 5, 9, and 100 PSCs, we conducted a constant photocurrent measurement and tracked the PCE output at the maximum power points (Figure 9c). The *n* = 9 devices demonstrated a stable PCE of 18.02%, indicating excellent operational stability in the illuminated environment. The *n* = 5 and 100 devices showcased stable PCE of 12.09% and 20.06%, respectively, indicating operational stability in the illuminated environment.

### 2.5. Stability Study of the Perovskite Layers and Devices

**Figure 10:**
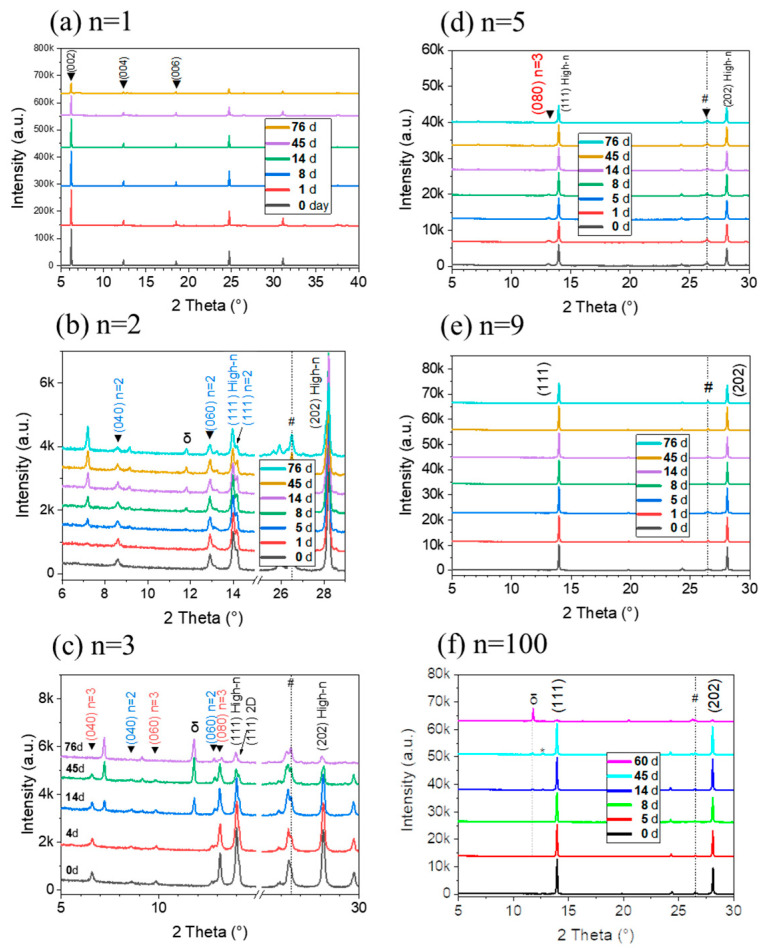
Changes in *θ-2θ* XRD patterns over time in ambient laboratory conditions for PMA_2_FA_*n*−1_Pb_*n*_I_3*n*+1_ for *n* = 1, 2, 3, 5, 9, and 100 layers. (Temperature: 15–28 °C, relative humidity (RH) 35–70%).

Introducing 2D materials into the perovskite structure is known to augment the layer resistance to air and moisture. The resilience of the layers against these stressors is a critical factor for the commercialization of PSCs. In Figure 10, the degradation of *n* = 1, 2, 3, 5, 9, and 100 layers under ambient conditions (temperature: 15–28 °C, relative humidity (RH) 35–70%) is tracked. After 76 days in the laboratory environment, the *n* = 5 and *n* = 9 samples still did not exhibit the δ-phase in the XRD pattern. The δ-phase appears in the *n* = 2, *n* = 3, and *n* = 100 samples XRD pattern after 8/14/8 days of aging, respectively.

Even after 76 days, the aged *n* = 9 perovskite layers did not show peak of the δ-phase and lead iodide, while the intensity of the perovskite peaks slightly decreased. This indicates that *n* = 9 perovskite layers exhibit significantly improved environmental stability. The enhanced air and humidity stability of the layers can be ascribed to the hydrophobicity of the large PMA^+^ cation that limits moisture ingress. By comparing the intensities of the perovskite (111) peak in XRD patterns, *n* = 9 was found to be the most stable sample (Figure 10).

The high stability of sample *n* = 9 was confirmed by measuring the layer UV/vis absorbance spectra (Figure S8). The changes in the absorption characteristics of the *n* = 1, 2, 3, 5, 9, and 100 samples further substantiated that the *n* = 9 film displays superior humidity stability compared to the other perovskite films. The absorption intensity of the *n* = 1, 2, 3, 5, and 100 perovskite films significantly decreased in comparison to the perovskite film of sample *n* = 9, signifying more or less degradation in the original layers under high humidity conditions. Conversely, the films of sample *n* = 9 retained a substantial portion of the initial absorption even after 45 days. The films of sample *n* = 9 showcase pure phases, large gains, leading to significantly enhanced humidity stability of the quasi-2D perovskite film.

**Figure 11:**
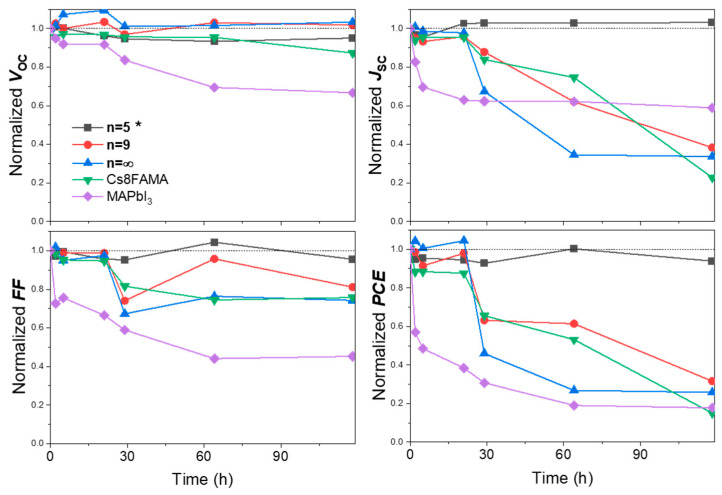
Changes in *J-V* curve parameters over time for PMA_2_FA_*n*−1_Pb_*n*_I_3*n*+1_ (*n* = 5 *, 9), the perovskite layers with * notation have been prepared with a 20% PbI_2_ excess and using 40 mol% of MACl additive. The curves of 3D perovskite (FA_1−x_MA_x_PbI_3_), 3D CsFAMA and 3D MAPbI_3_ perovskite solar cell aging are also presented. (Temperature: 15–28 °C, Relative Humidity (RH) 75–90%).

PSCs were prepared and stability tests of unencapsulated devices were conducted. We investigated three different 3D perovskites: MAPbI_3_, FA_1−x_MA_x_PbI_3_, and Cs_0.08_FA_0.92−x_MA_x_PbI_3_ and the *n* = 5 and *n* = 9 compounds that have been identified above as the most stable layers. The moisture resistance was examined by storing the unencapsulated cells under harsh condition: chamber with a high humidity of 75–90% RH, ambient light conditions and a temperature of 15–28 °C. Figure S9 shows the variation in the *J-V* curve parameters upon aging for 50 h. MAPbI_3_ was the less stable compound and the PCE decreased rapidly. The q2D devices *n* = 5 and *n* = 9 presented a deceiving stability and were not much more stable than the FA_1−x_MA_x_PbI_3_, and Cs_0.08_FA_0.92−x_MA_x_PbI_3_ PSCs. To address the poor q2D PSC stability, further PPS composition engineering was implemented, focusing on the *n* = 5 composition. We found that adding an excess of 20 mol% PbI_2_ and using 40 mol% MACl addive boosted the PCE to 17.32% (sample *n* = 5 * in Table 2). The *J-V* parameters were *V_oc_* = 1.091 V, J_sc_ = 23.28 mA/cm^2^, FF = 68.2%. These values are satisfactory compared with the data from the literature on low-n Ruddlesden–Popper PSCs presented in Table S1. Figure 11 shows the variation in the *J-V* curve parameters upon aging for 120 h. The 3D perovskites exhibited a poor stability, retaining less than 30% of the initial PCE after 120 h. The *n* = 9 device showed a slightly improved PCE evolution, but it was close to the triple cation 3D PSC. On the other hand, the *n* = 5 * one presented a very good stability and retained over 94% of its initial PCE after 120 h of aging under the selected harsh aging conditions. This is attributed to the suitable large PMA^+^ cation content which prevents the ingress of water molecules, resulting in significantly enhanced humidity stability of the PSCs.

## 3. Conclusions

We produced a series of 2D-RP perovskite films starting from solution with the PMA_2_FA_*n*−1_Pb_*n*_I_3*n*+1_ (*n* = 1, 2, 3, 5, 9, and 100) stoichiometry and containing a 36 mol% MACl additive. ^1^H NMR measurements have shown that the PMA^+^ spacer is partially eliminated upon the thermal annealing, and, consequently, the real *n* values in the final layers are higher than the *n* value in the PPS. The relative *n* enhancement raises with the initial *n* value. Our findings have revealed that the quasi-2D perovskite films (*n* = 2, *n* = 3, and *n* = 5) comprised mixed 2D phases, belonging to *n* = 2, *n* = 3, *n* = 4 and high-n phases. Fresh *n* = 100, *n* = 9, and *n* = 1 samples exhibited good purity. The larger the *n* values, the more uniform the perovskite layers are. The 2D phases with small *n* values tend to form at the bottom of the film, compromising the protection of the perovskite. We have followed the DMSO solvent elimination and determined the film growth direction using the GD-OES technique. Appropriate *n* values can yield solar cells with high efficiency and stability. The aging tests have shown that the sample with the initial *n* = 9 (corresponding to a final average *n* value of 84) is more stable than the others with *n* = 2, 3, 5, and 100. This sample exhibited a higher phase purity that is beneficial for stability and efficiency.

The highest PCEs of *n* = 9 and *n* = 100 PSCs reached 19.38% and 21.16%, respectively. Then, based on our comprehensive understanding of the system, we have focused on *n* = 5 and found that using MACl additive and an excess of PbI_2_ in the PPS leads to high power conversion efficiency (17.32%) and high stability q2D PSCs. This study represents a significant step forward in understanding the formation process of FA-based layered perovskites via one-step sequential deposition. By controlling the phase distribution, composition, and orientation, it offers a theoretical foundation and technical guidance for the preparation of q2D Ruddlesden–Popper perovskite solar cells.

## 4. Experimental Section

N,N-dimethylformamide (DMF, 99.8%), dimethyl sulfoxide (DMSO, 99.9%), isopropanol (IPA, 98%), 4-tert-butylpyridine (96%), acetonitrile (99.8%), chlorobenzene (CB, 99.8%) and lithium bis(trifluoromethanesulfonyl)imide (99.95%) were purchased from Sigma-Aldrich. Lead iodide (PbI_2_, 99.9985%), was purchased from TCI Europe N.V. TiO_2_ NR30-D paste, formamidinium iodide (FAI, 99.99%), phenylmethylammonium iodide (phenylmethylamine hydroiodide or benzylammonium iodide, PMAI 99.5%) and phenethylammonium iodide were bought from Greatcell Solar Materials. 2,2′,7,7′-tetrakis-(N,N-di-p-methoxyphenylamine)-9,9′-spirobifluorene (spiroOMeTAD, ≥99.8%) was bought from Borun New Materials Technology Ltd. All reagents were utilized in their original state without additional purification. Fluorine-doped SnO_2_ (FTO) coated glass substrates were TEC 7 from Pilkington. Methylammonium chloride (MACl, 99%), and Titanium (IV) isopropoxide were purchased from Alfa Aesar. Acetylacetone was procured from Merck.

The Fluorine-doped SnO_2_ (FTO) substrates (TEC 7 from Pilkington) were prepared as described in Ref. [40]. The TiO_2_ layers were prepared by spray pyrolysis and spin coating as in Ref. [31] The perovskite precursor solutions were formulated as PMA_2_FA_*n*−1_Pb_*n*_I_3*n*+1_ (*n* = 1, 2, 3, 5, 9, and 100) by dissolving precise stoichiometric quantities of PMAI, FAI, and PbI_2_ in a DMSO/DMF (volume ratio 1:4) mixed solution with a concentration of 1.2 M PbI_2_. Otherwise mentioned, 36 mol % MACl compared to FAI was employed as an additive. The solutions underwent initial stirring for a minimum of 2 h at room temperature in a N_2_-filled glovebox. Subsequently, 35 μL of this solution was deposited on top of the substrates. A two-step spin-coating regime was utilized: initially spinning at 1000 rpm for 10 s and then at 6000 rpm for 30 s. 100 µL of chlorobenzene was dripped 20 s after the initiation of the spinning process. The films were subsequently annealed on a hotplate at 150 °C for 15 min. The PEAI post-deposition treatment consisted of dropping 60 μL of a 10 mM 2-phenethylamine hydroiodide (PEAI) solution (2.49 mg in 1 mL of isopropanol) onto the cold perovskite film. A one-step spin-coating program was employed: 2000 rpm/s acceleration, 3000 rpm for 20 s. The spiro-OMeTAD layer and the gold back electrode were deposited as in Ref. [40].

The perovskite layers were characterized by XRD, GIWAXS, ^1^H NMR and GD-OES, spectrophotometry techniques as detailed elsewhere [31,33]. The solar cells were characterized by *J-V* curves measurements under standard AM 1.5G illumination using a calibrated Abet technologies Sun 2000 solar simulator and the EQE spectra were recorded by an Oriel QuantX-300 system [31]. More experimental details are given in the Supporting Information.

The full experimental details are provided in the Supporting Information.

## Figures and Tables

**Table 1:** Effect of *n* on the solvent elimination speed: Slopes, GI(%) parameter, and global growth direction.

System	Composition	SUS-2 ^a^	SLS-2 ^b^	GI ^c^ [%]	Growth Direction
(PMA)_2_FA_*n*−1_Pb_*n*_I_3*n*+1_	*n* = 5	−0.02653	−0.01093	+58.8	Downward
	*n* = 9	−0.01486	−0.01067	+28.2	Lateral
	*n* = 100	−0.01052	−0.01414	−25.6	Lateral

(^a^) SUS-2: The Slope of Upper layer in the Stage-2, (^b^) SLS-2: The Slope of Lower layer in the Stage-2, (^c^) GI (Gap Index) = (SUS-2 − SLS-2) × 100/Max{SUS-2,SLS-2}.

**Table 2:** *J-V* curve parameters of best PSC prepared from PMA_2_FA_*n*−1_Pb_*n*_I_3*n*+1_ (*n* = 1, 2, 3, 5, 9, and 100) precursor solutions.

Cell	Scan Direction	*Voc*/V	*Jsc* mA/cm^2^	*FF*/%	*PCE*/%	*HI*/%
*n* = 1	Reverse	0.785	1.53	44.91	0.53	/
	Forward	/	/	/	/	
*n* = 2	Reverse	0.779	8.93	41.45	2.88	/
	Forward	/	/	/	/	
*n* = 3	Reverse	0.885	10.83	30.93	2.96	/
	Forward	/	/	/	/	
*n* = 5	Reverse	1.081	20.81	61.29	13.78	14.9
	Forward	1.071	20.83	52.54	11.72	
*n* = 5 *	Reverse	1.091	23.28	68.20	17.32	10.1
	Forward	1.076	22.89	63.17	15.56	
*n* = 9	Reverse	1.022	24.72	76.72	19.38	14.7
	Forward	1.003	23.65	69.70	16.53	
*n* = 100	Reverse	1.023	25.68	80.56	21.16	8.1
	Forward	1.009	25.28	76.23	19.44	

* Optimized with 20% PbI_2_ excess and 40% MACl.

## Data Availability

Supplementary data are available in the Supporting Information. More data are available on demand to the corresponding author.

## Supplementary Materials

The following supporting information can be downloaded at: https://www.mdpi.com/article/10.3390/nano15100750/s1. Supplementary Experimental Section; Figure S1: Fabrication process and SEM top views of perovskite films; Figure S2: Evolution of chloride depth profile and peak integral; Figure S3: 1H NMR spectra and analysis; Figure S4: Ratio of (X_MFA_ + X_MA_)/X_FA_ measured by NMR; Figure S5: Investigated solar cell architecture and principle of GD-OES; Figure S6: Color change upon Stage 1; Figure S7: GD-OES profile analysis process; Figure S8: Changes in UV-Vis absorbance curves of perovskite films over time in ambient laboratory conditions; Figure S9: Changes in *J-V* curve parameters over time for PMA_2_FA_*n*−1_Pb_*n*_I_3*n*+1_ (*n* = 5, 9) solar cells; Table S1: Literature *J-V* curve parameters and PCEs data on Ruddlesden-Popper perovskite solar cells [10,29,41,42,43,44,45,46,47,48,49,50,51,52].
