# Supplementary material for: Spacer Loss upon 2D Ruddlesden–Popper Halide Perovskite Annealing Raises Film Properties and Solar Cell Performances
Source: Nanomaterials (Basel). 2025 May 16;15(10):750. doi: 10.3390/nano15100750 (PMC12113947; doi:10.3390/nano15100750)
Supplement: Supplementary file 1 [file nanomaterials-15-00750-s001.zip › nanomaterials-3615026-supplementary.pdf]

nanomaterials

MDPI

Supporting Information

Experimental Section

Preparation of the TiO2 layer

Fluorine-doped SnO_2 (FTO) substrates (TEC 7 from Pilkington) underwent etching using zinc powder and a 10 % HCl solution, followed by cleaning with soap and water. Subsequently, the substrates were immersed in a concentrated 2.2 M NaOH ethanol/water (10:1 v/v %) solution for 20 minutes and then rinsed with deionized water under ultrasonics for 15 minutes. The substrates were then subjected to heating at 500 °C for 15 minutes. The TiO_2 blocking compact layer (denoted as c-TiO_2) was fabricated using an aerosol spray pyrolysis technique, as detailed in the previous work of the research group. The resulting TiO_2 layer exhibited density, absence of pinholes, and a thickness of 30-35 nm. The TiO_2 paste for the preparation of the mesoporous layer (designated as meso-TiO_2) was pre-formulated and stirred for a minimum of 12 hours. The TiO_2 NR30-D paste (sourced from Greatcell) was diluted in ethanol at a ratio of 1:7 w/w. The mesoporous layer was prepared via spin coating by dispensing 45 μL of the prepared TiO_2 paste onto the compact TiO_2 layer, swiftly transferring it to a hotplate at 70 °C, and allowing it to dry for 5-10 minutes. Finally, the meso-TiO_2 underwent heating at 500 °C under an air flux for 30 minutes, followed by cooling to 200 °C, and removal from the hotplate. The thickness values were approximately 20 nm and 120-150 nm for the compact and mesoporous TiO_2 layers, respectively.

Preparation of 2D perovskite films with various n values

The precursor solutions were formulated as $PMA_2FA_{n-1}Pb_nI_{3n+1}$ (n = 1, 2, 3, 5, 9, and 100) by dissolving precise stoichiometric quantities of PMAI (282.1 mg, 141.2 mg, 94.0 mg, 56.5 mg, 31.3 mg and 2.8 mg), FAI (0 mg, 51.6 mg, 68.8 mg, 82.5 mg, 91.8 mg, 103.18 mg), and PbI_2 (276.6 mg) in a 100 μL/400 μL DMSO/DMF mixed solvent solution. The PbI_2 concentration was 1.2 M PbI_2. 36 mol % MACl (compared to FAI) (0 mg, 7.3 mg, 9.2 mg, 11.7 mg, 13.0 mg and 14.6 mg) was employed as an additive. The solutions underwent initial stirring for a minimum of 2 h at room temperature in a N_2 glovebox. Subsequently, 35 μL of this solution was deposited on top of the substrates. A two-step spin-coating regime was utilized: initially spinning at 1000 rpm for 10 s and then at 6000 rpm for 30 s. 100 μL of chlorobenzene was dripped 20 s after the initiation of the spinning process. The films were subsequently annealed on a hotplate at 150 °C for 15 min. The PEAI post-deposition treatment consisted of dropping 60 μL of a 10 mM 2-phenylethylamine hydroiodide (PEAI) solution (2.49 mg in 1 mL of isopropanol) onto the cold perovskite film. A one-step spin-coating program was employed: 2000 rpm/s acceleration, 3000rpm for 20s.

For the optimized n = 5* sample (with 20mol% PbI_2 excess and 40 mol% MACl), the PPS concentration was 1.08 M (PbI_2 concentration at 1.3 M) with: 50.9 mg PMAI, 74.51 mg FAI, 299.6 mg PbI_2 and 11.7 mg MACl (40 mol % MACl compared to FAI) in a 100 μL/400 μL DMSO/DMF mixed solvent solution.

Device manufacture

A new solution was prepared by dissolving 78 mg of spiro-OMeTAD in 1 mL of chlorobenzene. The spiro-OMeTAD layer doped with Cobalt (Co) was produced by incorporating 18 μL of tris(2-1H-pyrazol-1-yl)-4-tert-butylpyridine)-cobalt(III) tris(bis(trifluoromethylsulfonyl)imide) (376 mg in 1 mL acetonitrile) into the spiro-OMeTAD precursor

solution. The spiro-OMeTAD layer doped with lithium (Li) and Co was manufactured by adding 18 μL of Co-salt solution, 23 μL of bis(trifluoromethylsulfonyl)imide lithium salt solution (Li-TFSI) (517 mg in 1 mL ACN), and 39 μL of TBP (tert-butylpyridine) to the spiro-OMeTAD precursor solution. The substrates coated with the spiro-OMeTAD layer were transferred to a glovebox filled with nitrogen for overnight storage. The devices were then finalized by depositing an 80 nm thick gold contact in a vacuum evaporator.

Characterizations

The perovskite thin layer morphology was assessed using a field-emission SEM instrument (Zeiss Supra 40) in the in-lens mode. The structure of the organometal lead perovskite layers was evaluated with a PANanalytical X-Pert high-resolution X-ray diffractometer (XRD) operating at 40 kV and 45 mA, utilizing Cu Kα radiation with λ= 1.5406 Å. The layer's specular absorbance was determined using a Cary 5000 UV-Vis-NIR spectrophotometer. A glass/FTO/c-TiO_2/meso-TiO_2 sample was employed as the baseline. Photoluminescence spectra were obtained with a Cary Eclipse fluorescence spectrophotometer.

Glow-Discharge Optical Emission Spectrometry (GD-OES) analyses were conducted using a HORIBA Jobin Yvon GD Profiler 2 instrument. This apparatus featured an RF-generator (at 13.56 MHz), a standard HORIBA Jobin Yvon glow discharge source with a cylindrical anode of 4 mm inner diameter, and two optical spectrometers (a polychromator and a monochromator) for fast-optical detection. The Ar plasma was generated at an Ar pressure of 420 Pa and an applied power of 17 W. The precursor or perovskite film was positioned on an O-ring at one side of the plasma chamber and served as a cathode.

GIWAXS measurements were conducted using a microfocus X-ray source (IμS, Incoatec, Germany) emitting Cu Kα radiation (λ = 1.54 Å). GIWAXS patterns were captured with a 2D X–ray detector (Pilatus 100 K, Dectris, Switzerland) with an exposure time of 10 s. The incidence angle was set at 1°, and the distance between the sample and detector was 73 mm.

10 perovskite layers deposited on sprayed c-TiO_2 substrates were dissolved in 0.75 mL DMSO-d6 solvent. ^{1}H NMR spectra were recorded on a Bruker Neo 500 spectrometer, using DMSO-d6 as solvent. Chemical shifts were reported in ppm downfield from tetramethylsilane and were referenced to the residual hydrogen signal of deuterated solvent (2.50 ppm). ^{1}H spectra were acquired with a 90° pulse of 10.0 μs, 64K data points over a 10 kHz spectra width, an acquisition time of 3.28 s, a recycle delay of 10 s and 256 scans. The Fourier transform was applied to the FID without prior exponential line-broadening. For each spectrum, the integrations were normalized to the residual DMSO-d6 proton signal used as an internal reference (set at 100 for normalization), allowing comparison of the integrations of the different spectra.

The integration bounds on either side of each NMR peak were the same from one spectrum to another with a +/− 5 Hz margin of error. Integrations were given with an accuracy of +/− 1% for peaks with a signal-to-noise ratio greater than 10% and 5% for peaks with an S/N greater than 1.

The *J-V* curves were obtained using a Keithley 2410 digital sourcemeter, employing a voltage scan rate of 0.1 $V.s^{-1}$. Solar cells were illuminated with a solar simulator (Abet Technology Sun 2000) filtered to replicate AM 1.5G conditions (100 mW/cm^2). The illuminated surface was defined by a black mask with an aperture diameter of 3 mm. The power density was calibrated at 100 $mW.cm^{-2}$ using a reference silicon solar cell. Tracking experiments were conducted under ambient conditions, and the current was monitored at the voltage of maximum power. External quantum efficiency (EQE) spectra were recorded using an Oriel QUANTX-300 system.

For the stability test, the perovskite films were placed in an ambient condition (temperature is 15-28°C, relative humidity (RH) is 35-70%) and were collected for UV vis and

XRD measurements after several days. The PSCs were stored in a high RH cabinet (15-28°C, RH75-90%) to accelerate the aging.

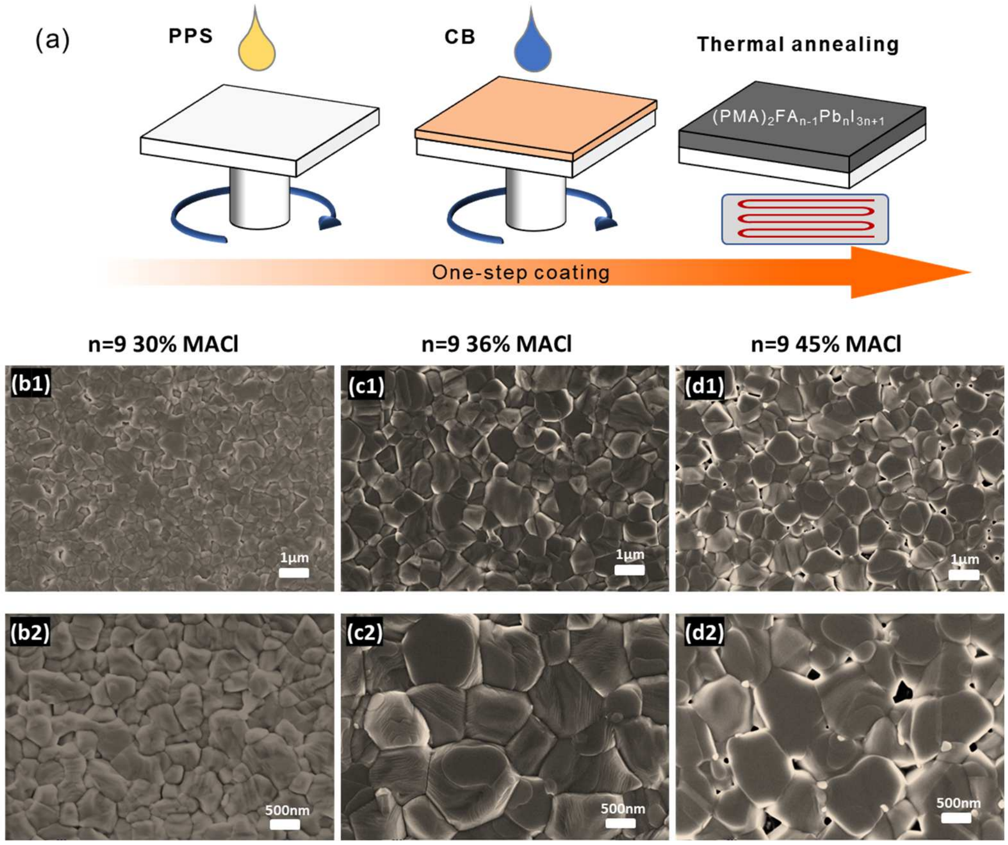

Figure S1. (a) Schematic representation of the fabrication process of FA-based 2D RP perovskite solar cells using the one-step method. (b-d) Effect of MACl additive concentration on the perovskite film morphology. SEM top-view pictures of 2D $PMA_2FA_8Pb_9I_{28}$ perovskite layers (n = 9): (b) 30% MACl additive; (c) 36% MACl additive; (d) 45% MACl additive.

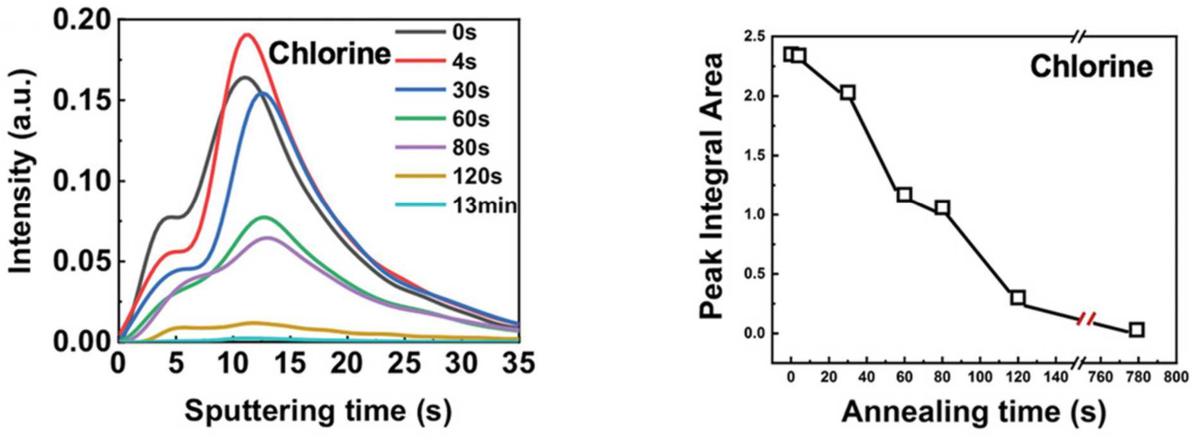

Figure S2. (a) Evolution of chloride depth profile in a $(PMA)_2FA_{n-1}Pb_nI_{3n+1}$ (n = 100) layer upon thermal annealing. (b) Corresponding peak integral area evolution.

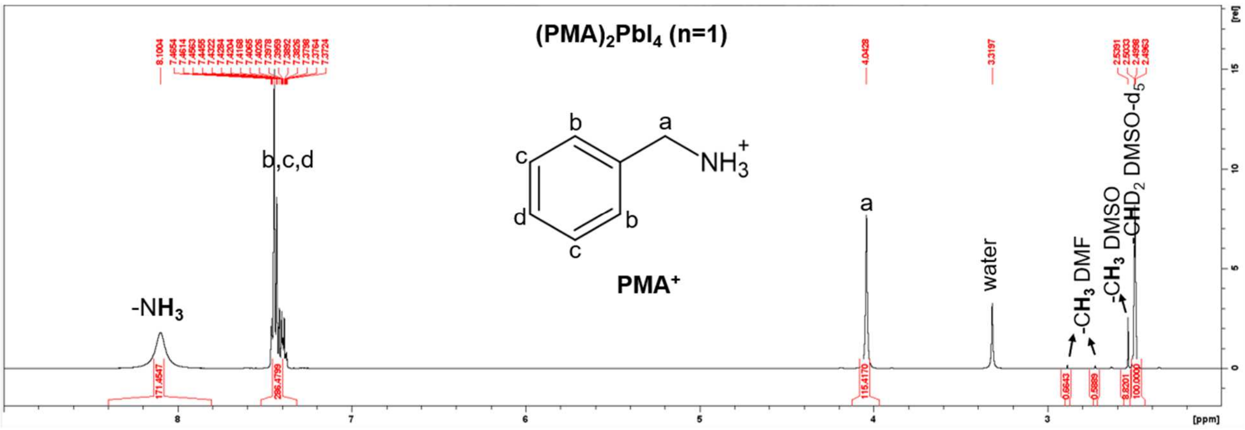

Species	Identification	Chemical shift (ppm)	Integration reported over 1 H	X_{cation} (%)
**PMA⁺**	Ha	4.04	**57.71**	**100**
	Hb + Hc + Hd	7.47 – 7.37	57.30	
**DMSO-d5**	-CHD$_2$	2.50	N/A	N/A
**DMSO**	-CH$_3$	2.54	N/A	N/A
**DMF**	-CH$_3$	2.73<br>2.88	N/A	N/A

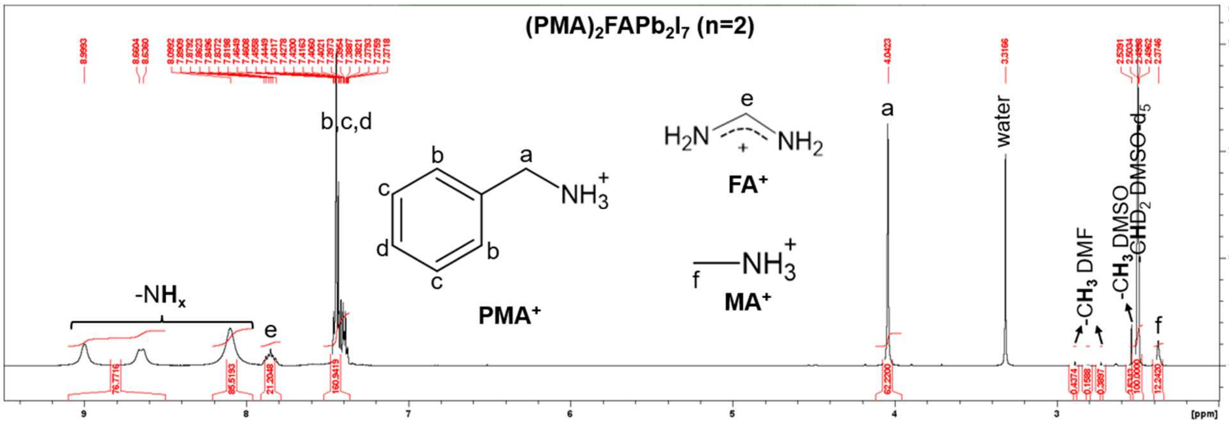

Species	Identification	Chemical shift (ppm)	Integration reported over 1 H	X_{cation} (%)
**PMA⁺**	Ha	4.04	**30.88**	**55.3**
	Hb + Hc + Hd	7.47 – 7.37	29.76	
**FA⁺**	He	7.84	**21.02**	**37.6**
**MA⁺**	Hf	2.37	**3.90**	**7.0**
**3MFA⁺**	-CH$_3$	2.81	**0.05**	**0.1**
**DMSO-d5**	-CHD$_2$	2.50	N/A	N/A
**DMSO**	-CH$_3$	2.54	N/A	N/A
**DMF**	-CH$_3$	2.73<br>2.88	N/A	N/A

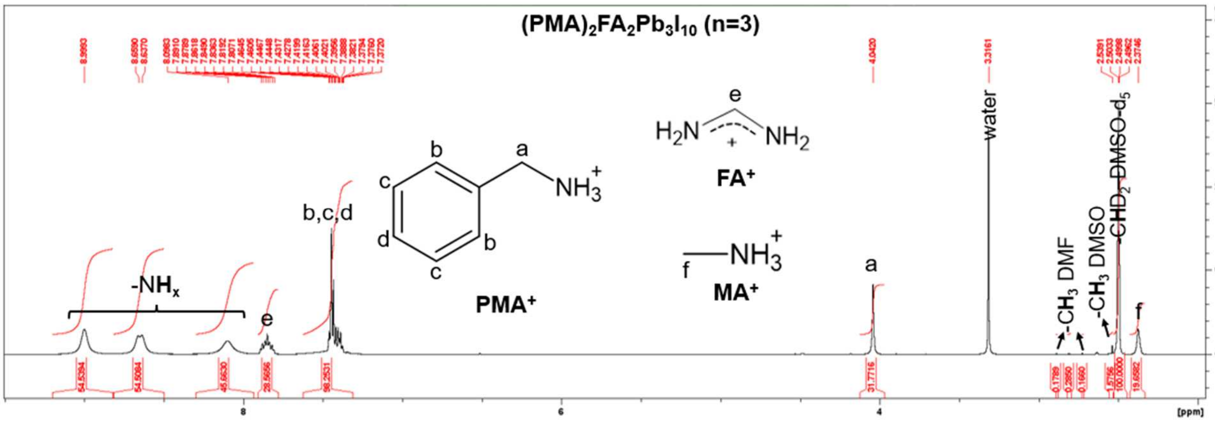

Species	Identification	Chemical shift (ppm)	Integration reported over 1 H	X_{cation} (%)
PMA^+	Ha	4.04	**15.89**	**31.3**
	Hb + Hc + Hd	7.47 – 7.37	19.65	
FA^+	He	7.84	**28.57**	**56.2**
MA^+	Hf	2.37	**6.28**	**12.3**
$3MFA^+$	$-CH_3$	2.81	**0.10**	**0.2**
DMSO-d5	$-CHD_2$	2.50	N/A	N/A
DMSO	$-CH_3$	2.54	N/A	N/A
DMF	$-CH_3$	2.73<br>2.88	N/A	N/A

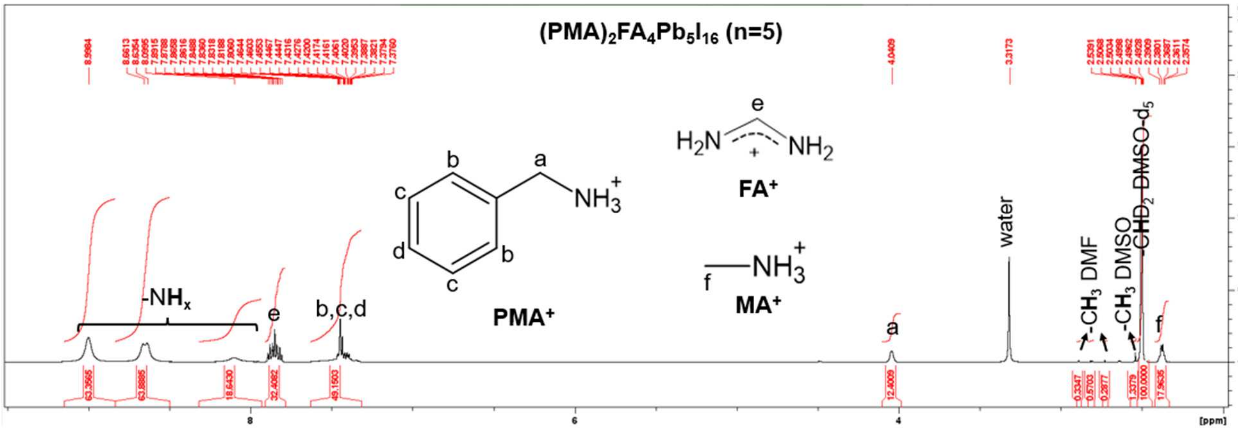

Species	Identification	Chemical shift (ppm)	Integration reported over 1 H	X_{cation} (%)
PMA^+	Ha	4.04	**6.20**	**13.9**
	Hb + Hc + Hd	7.47 – 7.37	6.38	
FA^+	He	7.84	**32.41**	**73.0**
MA^+	Hf	2.37	**5.75**	**12.9**
$3MFA^+$	$-CH_3$	2.81	**0.10**	**0.2**
DMSO-d5	$-CHD_2$	2.50	N/A	N/A
DMSO	$-CH_3$	2.54	N/A	N/A
DMF	$-CH_3$	2.73<br>2.88	N/A	N/A

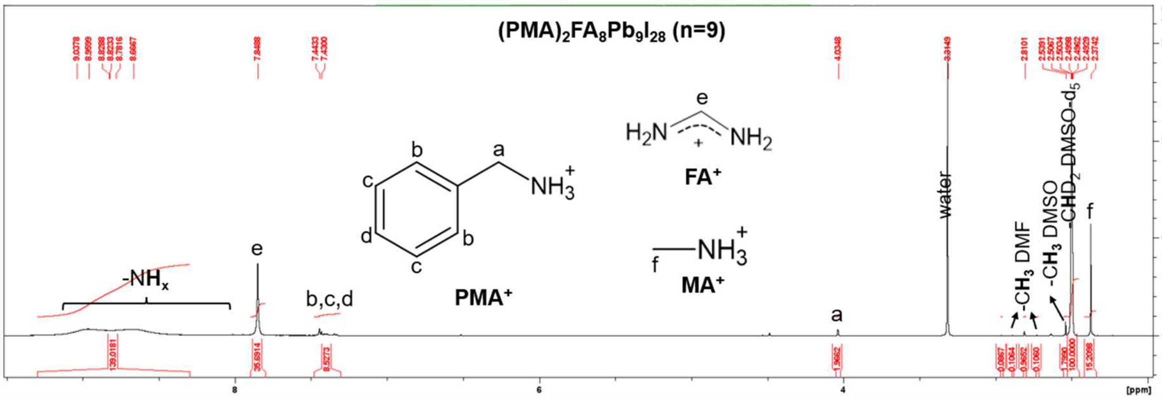

Species	Identification	Chemical shift (ppm)	Integration reported over 1 H	X_{cation} (%)
PMA+	Ha	4.04	**0.98**	**2.4**
	Hb + Hc + Hd	7.47 – 7.37	0.59	
FA+	He	7.84	**35.66**	**85.2**
MA+	Hf	2.37	**4.82**	**11.5**
3MFA+	$-CH_3$	2.81	**0.32**	**0.8**
1MFA+	$-CH_3$	2.96	**0.03**	**0.1**
DMSO-d5	$-CHD_2$	2.50	N/A	N/A
DMSO	$-CH_3$	2.54	N/A	N/A
DMF	$-CH_3$	2.73<br>2.88	N/A	N/A

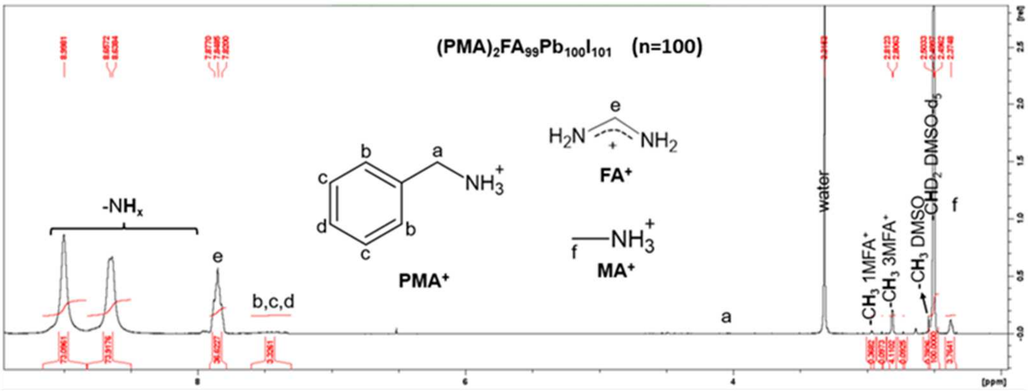

Species	Identification	Chemical shift (ppm)	Integration reported over 1 H	X_{cation} (%)
PMA+	Ha	4.04	**0.00**	**0.0**
	Hb + Hc + Hd	7.47 – 7.37	0.00	
FA+	He	7.84	**36.50**	**93.6**
MA+	Hf	2.37	**1.02**	**2.6**
3MFA+	$-CH_3$	2.81	**0.32**	**3.5**
1MFA+	$-CH_3$	2.96	**0.03**	**0.3**
DMSO-d5	$-CHD_2$	2.50	N/A	N/A
DMSO	$-CH_3$	2.54	N/A	N/A
DMF	$-CH_3$	2.73<br>2.88	N/A	N/A

Figure S3. ^1H NMR spectra (DMSO-d6) of $((PMA)_2FA_{n-1}Pb_nI_{3n+1}$ (n = 1, 2, 3, 5, 9 and 100) perovskite films. The films were annealed at 150 °C for 15 min.

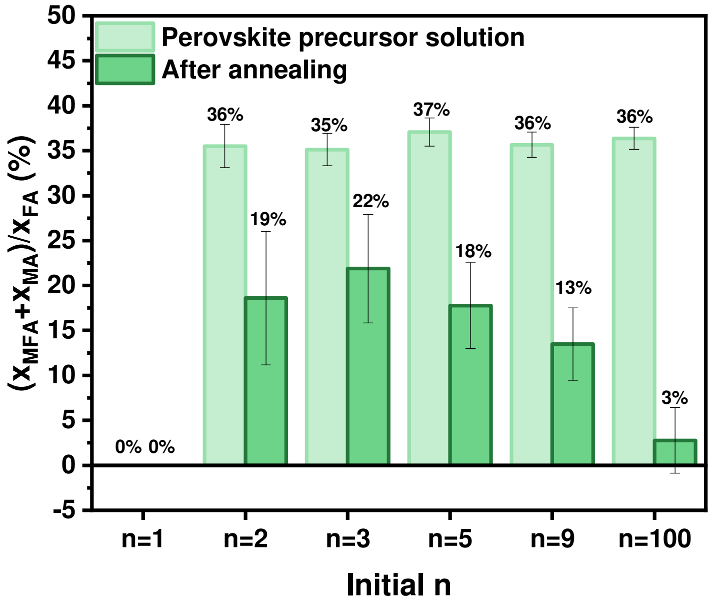

Figure S4. Ratio of $(X_{MFA} + X_{MA})/X_{FA}$ measured by NMR in the $((PMA)_2FA_{n-1}Pb_nI_{3n+1}$ (n = 1, 2, 3, 5, 9 and 100) with 36% MACl in the PPSs (light green) and in the perovskite film annealed at 150 °C for 15 min (dark green).

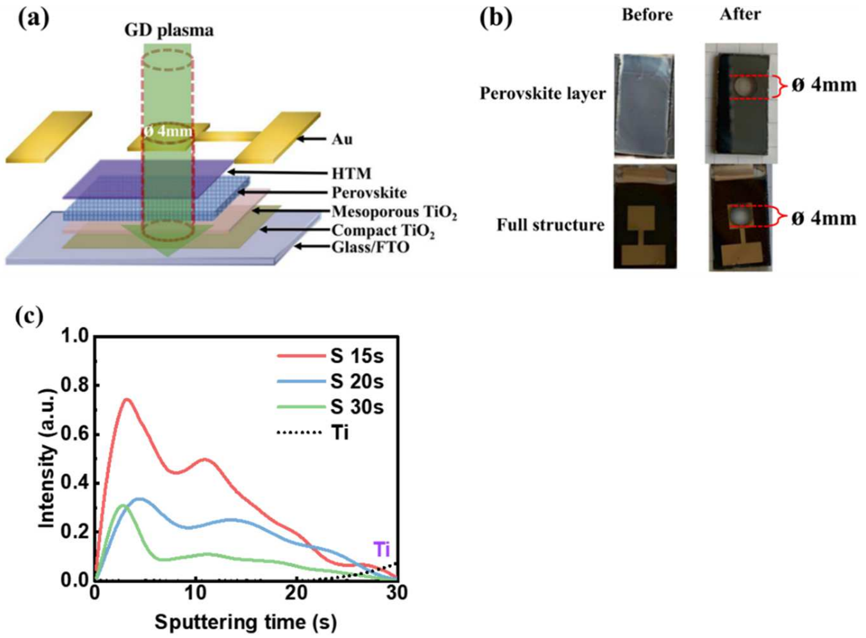

Figure S5. (a) Investigated solar cell architecture with the scale of the GD plasma beam passing through the perovskite solar cell (the detection diameter is 4 mm, HTM is the hole transporting material). (b) Perovskite film and full device before and after their investigation by GD-OES. (c) Example of evolution of GD-OES sulfur element (S) profile upon thermal annealing. The dashed line is the titanium element profile.

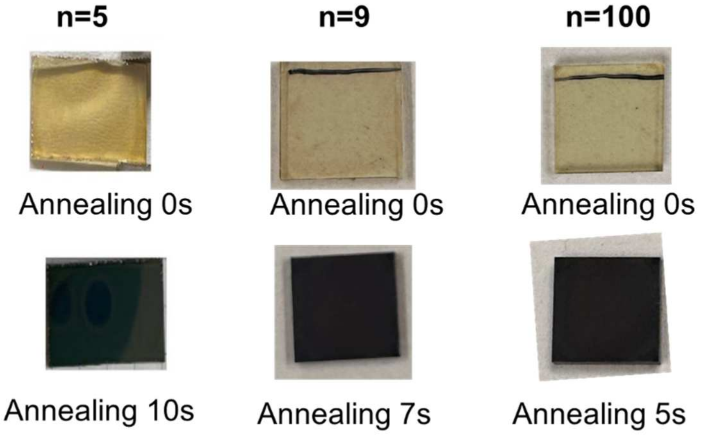

Figure S6. Color change upon Stage-1. $(PMA)_2FA_{n-1}Pb_nI_{3n+1}$ (n = 5, 9 and 100) film after spin-coating and at the color darkening time.

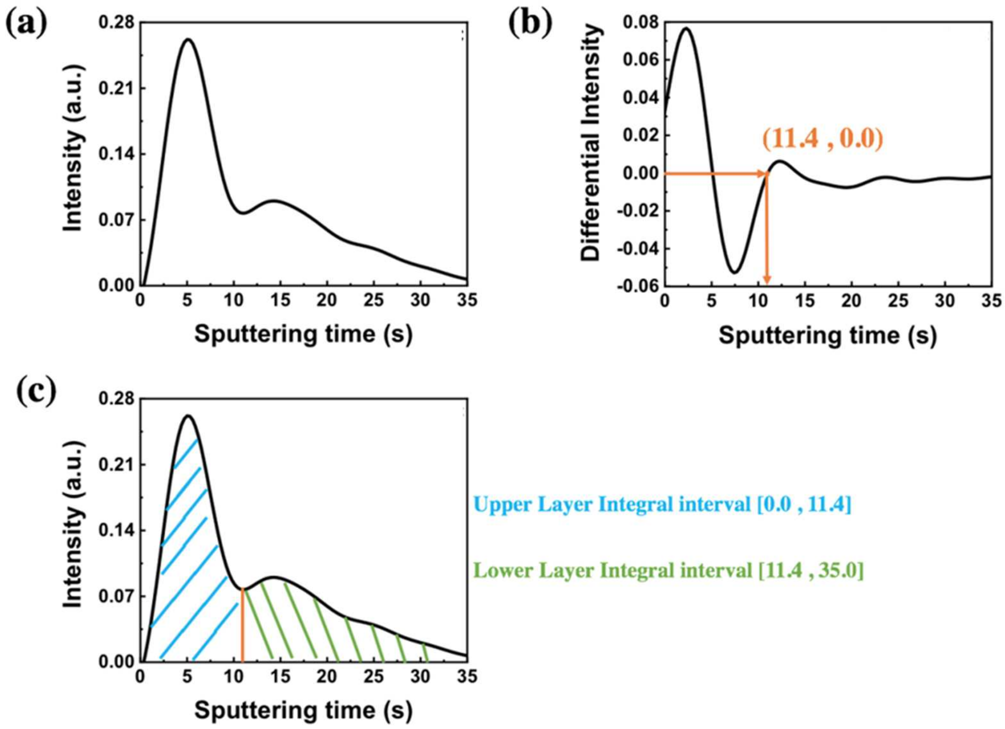

Figure S7. GD-OES profile analysis process. (a) GD-OES sulfur element (S) profile in a perovskite precursor layer. (b) Differential data of (a). (c) Distribution of upper layer and lower layer in (a).

Figure S7 depicts the integrals determination for the upper layer and the lower layers. For each film at different annealing durations, we have reported in **Figure 6(g-i)** the integrals for the two sublayers. We found that (re)crystallization occurred in Stage-1, while layer growth predominantly occurred in Stage-2. Throughout Stage-2, we observed the consistent solvent removal and calculated the slopes of two linear fits: SUS-2 slope for the upper layer and SLS-2 slope for the lower layer. Subsequently, we defined a parameter known as GI, standing for Gap Index, which correlates with the relative growth speed of each layer segment:[35]

$$GI(\%) = (SUS\text{-}2 - SLS\text{-}2) * 100 / Max\{SUS\text{-}2, SLS\text{-}2\} \quad (1)$$

By examining and contrasting the slopes of the two curves (**Table 1**), we can precisely deduce the direction of layer growth for various perovskites. As stated in Ref.[35]. GI serves as the principal parameter for gauging distinctions in layer growth among perovskites.

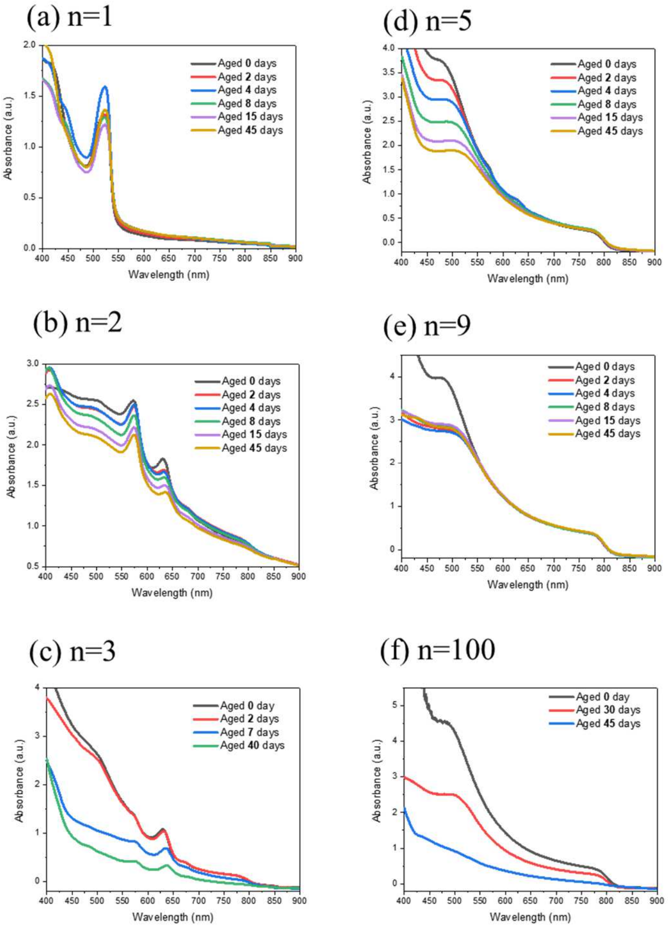

Figure S8. Changes in UV-Vis absorbance curves over time in ambient laboratory conditions for $PMA_2FA_{n-1}Pb_nI_{3n+1}$ for n = 1, 2, 3, 5, 9, and 100 layers. (Temperature: 15-28°C, relative humidity (RH) 35-70%).

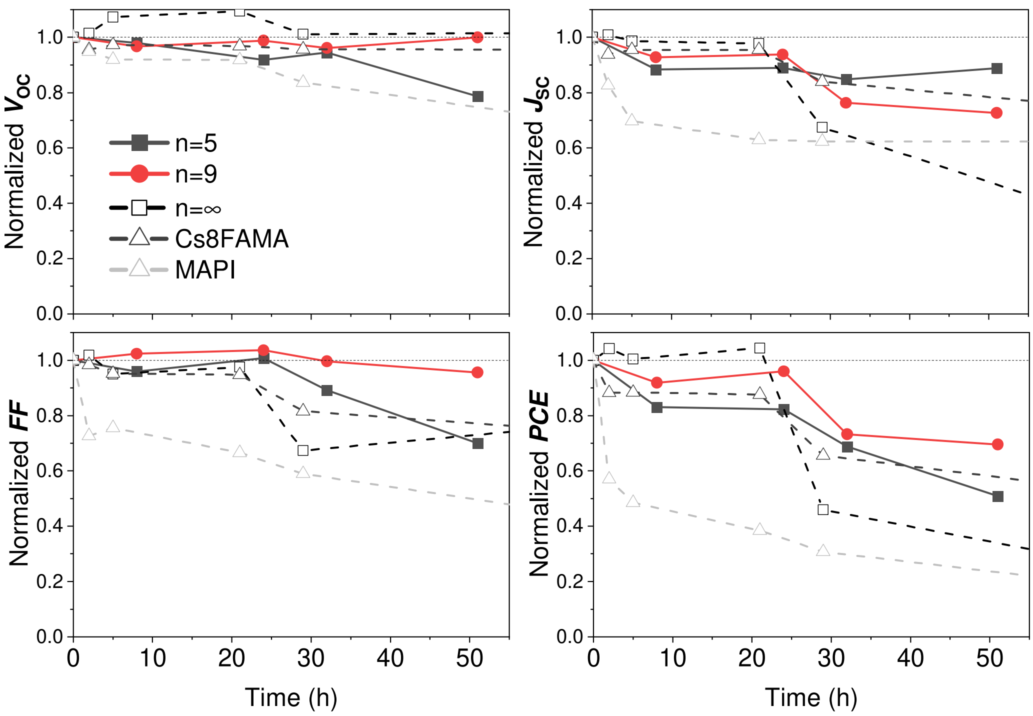

Figure S9. Changes in *J-V* curve parameters over time for $PMA_2FA_{n-1}Pb_nI_{3n+1}$ (n = 5, 9) solar cells. The curves of 3D perovskite ($FA_{1-x}MA_xPbI_3$) (n = ∞), 3D CsFAMA and 3D $MAPbI_3$ perovskite solar cell aging are also presented. (Temperature: 15-28°C, Relative Humidity (RH) 75-90%).

Table S1. Literature *J-V* curve parameters and PCEs data on Ruddlesden-Popper perovskite solar cells with the employed spacer, perovskite claimed composition and strategies employed.

Active layer*	J_{SC} [mA cm^{-2}]	V_{OC} [V]	FF [%]	PCE [%]	Strategy	Refs.
$(FPEA)_2FA_4Pb_5I_{16}$	22.24	1.18	79.53	21.07	MACl additive	[41]
$(CNBTh)_2MA_4Pb_5I_{16}$	21.67	1.18	81.20	20.82	Donor-acceptor (D-A) structure of the spacer	[42]
$(GA)_2MA_4Pb_5I_{16}$	21.64	1.19	79.67	20.44	NH_4SCN, MACI additive	[43]
$(HA)_2FA_3Pb_4I_{13}$	23.60	1.09	77.86	20.03	Selenourea additive	[44]
$(3,3\text{-}DFAz)_2(MA_{0.95}FA_{0.05})_3Pb_4(I_{13-x}Cl_x)$	22.28	1.11	80.26	19.85	Fluorinated dipole spacer	[45]
$(PMA)_2FA_5Pb_6I_{19}$	24.57	1.10	71.58	19.39	MACl additive, 20% PbI2 excess, 4F-PEAI treatment	[46]
$(AA)_2MA_4Pb_5I_{16}$	19.91	1.22	78.56	19.08	Sulfonium-cations-assisted strategy	[47]
$(PEA)_2MA_3Pb_4I_{13}$	18.52	1.20	83.39	18.48	$NH_4I_{0.2}Cl_{0.8}$	[48]
$(3BBA)_2MA_2Pb_3I_{10}$	18.22	1.23	81.2	18.20	MACI additive	[49]

$(PEA)_2MA_4Pb_5I_{16}$	17.91	1.22	82.4	18.04	Vacuum poling	[50]
$BA_2MA_2Pb_3I_{10}$	15.12	1.30	76.53	15.04	DMSO atmosphere	[51]
$(BA)_2MA_3Pb_4I_{13}$	19.95	1.08	63.47	13.68	Cs doping	[52]
$(BA)_2MA_3Pb_4l_{13}$	16.76	1.01	74.13	12.52	Hot-casting	[53]
$(PEA)_2MA_2Pb_3I_{10}$	6.72	1.18	60	4.73	Spin-coating	[54]

*FPEA: 4-fluorophene thylammonium; GA: guanidinium; HA: hexylamine; PMA: phenylmethylammonium; AA: amylammonium; PEA: phenethylammonium; 3BBA: 3-bromobenzylammonium; BA: butylammonium.
